# Proteomic landscape of astrocytes and pericytes infected with HIV/SARS-CoV-2 mono/co-infection, impacting on neurological complications

**DOI:** 10.21203/rs.3.rs-3031591/v1

**Published:** 2023-06-12

**Authors:** Arpan Acharya, Anoop T Ambikan, Michellie Thurman, Mohid Reza Malik, Shetty Ravi Dyavar, Ákos Végvári, Ujjwal Neogi, Siddappa N Byrareddy

**Affiliations:** University of Nebraska Medical Center; Karolinska Institutet; University of Nebraska Medical Center; University of Nebraska Medical Center; University of Nebraska Medical Center; Karolinska Institutet; Karolinska Institutet; University of Nebraska Medical Center

**Keywords:** SARS-CoV-2, HIV, Co-infection, COVID-19, ACE-2, HAND, LONG-Covid, PASC, Astrocytes and Pericytes

## Abstract

**Background:**

Although most individuals recover from coronavirus disease 2019 (COVID-19) within a few weeks, some people continue to experience a wide range of symptoms known as post-acute sequelae of SARS-CoV-2 (PASC) or long COVID. Majority of patients with PASC develop neurological disorders like brain fog, fatigue, mood swings, sleep disorders, loss of smell and test among others collectively called neuro-PASC. While the people living with HIV (PWH) do not have a higher risk of developing severe disease and mortality/morbidity due to COVID-19. As a large section of PWH suffered from HIV-associated neurocognitive disorders (HAND), it is essential to understand the impact of neuro-PASC on people with HAND. In pursuit of this, we infected HIV/SARS-CoV-2 alone or together in primary human astrocytes and pericytes and performed proteomics to understand the impact of co-infection in the central nervous system.

**Methods:**

Primary human astrocytes and pericytes were infected with SARS-CoV-2 or HIV or HIV + SARS-CoV-2. The concentration of HIV and SARS-CoV-2 genomic RNA in the culture supernatant was quantified using reverse transcriptase quantitative real time polymerase chain reaction (RT-qPCR). This was followed by a quantitative proteomics analysis of mock, HIV, SARS-CoV-2, and HIV + SARS-CoV-2 infected astrocytes and pericytes to understand the impact of the virus in CNS cell types.

**Results:**

Both healthy and HIV-infected astrocytes and pericytes support abortive/low level of SARS-CoV-2 replication. In both mono-infected and co-infected cells, we observe a modest increase in the expression of SARS-CoV-2 host cell entry factors (ACE2, TMPRSS2, NRP1, and TRIM28) and inflammatory mediators (IL-6, TNF-α, IL-1β and IL-18). Quantitative proteomic analysis has identified uniquely regulated pathways in mock vs SARS-CoV-2, mock vs HIV + SARS-CoV-2, and HIV vs HIV + SARS-CoV-2 infected astrocytes and pericytes. The gene set enrichment analysis revealed that the top ten enriched pathways are linked to several neurodegenerative disorders, including Alzheimer’s disease, Parkinson’s disease, Huntington’s disease, and amyotrophic lateral sclerosis.

**Conclusions:**

Our study emphasizes the significance of long-term monitoring of patients co-infected with HIV and SARS-CoV-2 to detect and understand the development of neurological abnormalities. By unraveling the molecular mechanisms involved, we can identify potential targets for future therapeutic interventions.

## Introduction

Three years after the outbreak of the Coronavirus disease 2019 (COVID-19) pandemic caused by the severe acute respiratory syndrome coronavirus 2 (SARS-CoV-2), the spread of the virus continues its catastrophic impact on people’s lives [1–3]. Despite COVID-19 being classified primarily as a respiratory illness and later as a disease of the vasculature [4]. The impact of coronaviruses in the central and peripheral nervous system (CNS/PNS) is well established lending from previous coronavirus outbreaks like SARS-CoV in 2003 and MERS-CoV in 2009 [5–8]. The early onset of neurological symptoms during acute COVID-19 indicates severe disease outcomes associated with higher mortality and morbidity rates [9]. Along with that, the development of cerebrovascular diseases is widely observed among COVID-19 patients having other co-morbid conditions like ageing, hypertension, and type 2 diabetes [10].

The post-acute neurologic sequelae of COVID-19 are associated with headache, vertigo, anosmia, tinnitus, and dysautonomia, among others [11]. Cognitive impairment is also common among hospitalized and non-hospitalized patients long after recovery from the active disease, substantially disrupting the standard of living.

The impact of SARS-CoV-2 infection among people living with human immunodeficiency virus (PWH) has been a matter of concern during the COVID-19 pandemic due to their compromised immune system and the overlapping co-morbid conditions with COVID-19 [12, 13]. Surprisingly, multiple clinical cohort studies of HIV-1/SARS-CoV-2 co-infected persons reported that PWH does not pose a higher risk of severe disease outcomes and mortality/morbidity [14]. A large section of PWH suffered from mild neurocognitive impairment which leads to depression, mood swings and memory loss [15]. HIV-associated chronic inflammation of the CNS is a major contributor to the development of HIV-Associated Neurocognitive Disorder (HAND), but the exact molecular mechanism of the disease pathogenesis remains obscure [16, 17]. While the lung pathogenesis of COVID-19 has been studied widely, the pathophysiology and the underlying mechanism of SARS-CoV-2-associated neurological disorders remain understudied. In postmortem brain tissue from COVID-19 patients, the presence of viral dsRNA, T-cell and macrophage infiltration, and associated perivascular inflammation, as well as compromised blood-brain barrier integrity were observed [18]. Single-nucleus RNA sequencing (snRNA-seq) analysis of the dorsolateral prefrontal cortex, medulla oblongata, and choroid plexus from severe COVID-19 patients’ postmortem brain tissue indicates altered cellular pathways associated with cell activation, mobility, and phagocytosis without detectable SARS-CoV-2 [19]. Using SARS-CoV-2 infected astrocytes in-vitro and a hamster model of COVID-19, it was found that SARS-CoV-2 impact the brain’s central carbon metabolic pathways and synaptic transmissions due to neurochemical imbalance. This phenomenon was commonly observed in several neurodegenerative disorders like Huntington’s disease and Parkinson’s disease and as such were proposed this as probable mechanism of COVID-19 associated cognitive impairments [20]. It was also reported that spike protein of SARS-CoV-2 dysregulates the vascular and immune function of brain pericytes responsible for a cerebrovascular disorder associated with COVID-19 [21]. Using 3T-MRI, the presence of orbitofrontal cortical atrophy was observed, which is correlated with anxiety and cognitive disorders among mildly infected COVID-19 patients [22]. Another study reported olfactory neuroepithelium as a hub of SARS-CoV-2 infection and associated inflammation and the presence of viral genetic materials within the olfactory mucosa of long-COVID patients with anosmia [23]. However, the short- and long-term impacts of SARS-CoV-2 on the CNS of PWH, who already have a compromised immune system and fragile blood brain barrier (BBB), remains elusive.

Due to the challenges associated with studying the CNS pathogenesis among HIV/SARS-CoV-2 co-infected people, herein we present an *invitro*, HIV/SARS-CoV-2 co-infection model in primary astrocytes and pericytes as these are the primary target cells for SARS-CoV-2 within the CNS as described above [18, 24]. Both the cell types support abortive SARS-CoV-2 replication. However, quantitative proteomics and subsequent gene ontology pathway enrichment analysis of differentially up/down regulated proteins between different groups reveled that out of the top ten enriched pathways many are associated with the development of neurodegenerative disorders like Alzheimer’s Disease (AD), Parkinson’s Disease (PD), Huntington Disease (HD) and Amyotrophic lateral sclerosis (ALS) in SARS-CoV-2 and HIV + SARS-CoV-2 infected astrocytes and pericytes when compared to mock and only HIV infected cells. This indicates possible involvement astrocytes and pericytes in the development of neurological disorders in COVID-19 patients and possible aggravation of HIV Associated Neurological Disorders (HAND) in SARS-CoV-2 infected people living with HIV.

## Material and Methods

### Cells

In this study, we used human brain microvascular astrocytes (catalog # HMP202; Neuromics) and pericytes (Catalogue # HMP104; Neuromics) isolated from normal human brain cortical tissue. The cells were grown in T25 flasks using an astrocyte-growth medium (Catalogue # PGB003; Neuromics) and a Pericyte-Growth medium (Catalogue # PGB001; Neuromics). The media was changed every two days, and cells were sub-cultured at a ratio of 1:2 every 7 days. Calu-3 cells (a human lung adenocarcinoma cell line) were cultured in Eagle’s Minimum Essential Medium (EMEM) (ATCC 30-2003) containing 10% FBS and used for growing the viral stock. Vero-E6 cells (an African green monkey kidney cell line) were cultured in DMEM containing 10% fetal bovine serum (FBS), 2 mM L-glutamine, 100 units/ml penicillin, 100 units/ml streptomycin, 10 mM HEPES (pH 7.4) and used for viral titer estimation.

### Production and titration of SARS-CoV-2 and HIV-1_ADA_ stocks

SARS-CoV-2 isolate USA-WI1/2020 (BEI; cat# NR-52384) was passaged in Calu-3 cells for generating viral stock. The viral titer was determined using the plaque assay as described by us previously [25]. HIV-1ADA, a laboratory-adapted M-tropic HIV-1 strain, was grown in human monocyte-derived macrophages (MDM) and tittered by RT-activity assay as described earlier [26].

### Virus infection of astrocytes and pericytes

**Figure 1:**
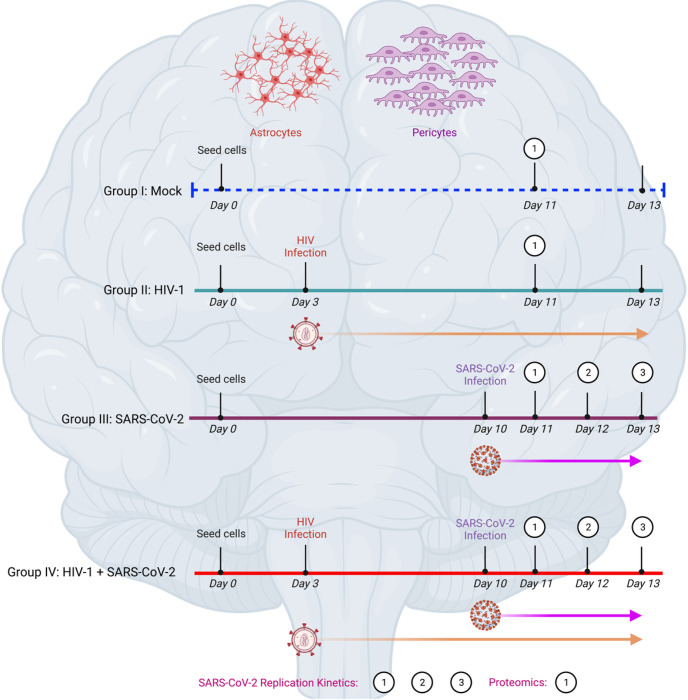
Experimental outline of the study. In this study, we used human brain microvascular astrocytes and pericytes isolated from normal human brain cortical tissue. There were four groups included in the study for each cell type. Group I: Mock (uninfected controls; these cells were seeded on day 0 and harvested on day 11 for proteomic analysis); Group II: HIV-1 (these cells were seeded on day 0, infected with HIV-1 on day 3 and harvested on day 11 for proteomic analysis); Group III: SARS-CoV-2 (these cells were seeded on day 0, infected with SARS-CoV-2 on day 10 and harvested on day 11 for proteomic analysis and on day 11, 12 and 13 for SARS-CoV-2 replication kinetics evaluation); Group IV: HIV-1+SARS-CoV-2 (these cells were seeded on day 0, infected with HIV-1 on day 3, infected with SARS-CoV-2 on day 10 and harvested on day 11 for proteomic analysis and on day 11, 12 and 13 for SARS-CoV-2 replication kinetics evaluation). Astrocytes and pericytes from three healthy donners were used in all experiments.

The astrocytes and pericytes were divided into 4 four experimental groups as described in Fig. 1. For groups II and IV, the astrocytes and pericytes were infected with 0.1 multiplicity of infection (MOI) of HIV-1ADA. The viral inoculum was added to the cells and incubated at 37° C for 4 h. After that, the cell free virus was removed by washing 3 times with 1X PBS and replenished with fresh media. Culture media was collected at designated time points for measuring the HIV-1 RNA copies using qPCR assay [27]. For groups III and IV, the cells were infected with 1.0 MOI of SARS-CoV-2, as described earlier [28]. Culture supernatant was collected at 24, 48 and 72 hours post infection (hpi). The SARS-CoV-2 viral load was quantified in the culture supernatant using RT-QPCR as described previously [25].

### Quantitative proteomics analysis

Proteomics analysis of SARS-CoV-2 and/or HIV-1 infected astrocytes and pericytes was performed as described previously (Appelberg et al., 2020). In brief, 24 h post-infection, the cells were harvested, washed with ice-cold 1X PBS, and proteins were extracted using an SDS-based buffer mixed with Protease and phosphatase inhibitor cocktails (Thermo Fisher SCIENTIFIC; cat # 78430). This was followed by the digestion of the proteins on S-Trap micro columns (Protifi, USA) and then the resulting peptides were labeled with isobaric TMTpro^™^ reagents. Then, concatenate the labeled peptides following high pH (HpH) reversed-phase chromatographic fractionation into 12 fractions. Peptides in each fraction were separated by low pH reversed-phase chromatography on an Ultimate 3000 UHPLC (Thermo Fisher Scientific, USA) in a 120 min linear organic modifier gradient. Data acquisition was performed on a Fusion Lumos tribrid Orbitrap mass spectrometer (Thermo Fisher Scientific, USA) in data-dependent acquisition (DDA) mode, isolating the most intense precursors in 2 s at 120,000 mass resolution in the mass range of *m/z* 350–1500, and applying maximum injection time (IT) of 50 ms and dynamic exclusion of 45 s; precursor isolation width of 0.7 Th with high collision energy (HCD) of 34% at a resolution of 50,000 and maximum IT of 86 ms in MS2 event.

Proteins were searched against SwissProt human databases using the search engine Sequest HT in Proteome Discoverer v2.4 (ThermoFisher Scientific, USA) software environment allowing a maximum of two missed cleavages. Oxidation of methionine, deamidation of asparagine and glutamine, and TMTpro modification of lysine and N-termini were set as variable modifications, while carbamidomethylation of cysteine was used as fixed modification. The false discovery rate (FDR) was set to 1%.

### Proteomic data analysis

Differential expression analysis was performed using the R package limma v3.50.0 [29]. Proteins with p.adj < 0.05 were considered significantly expressed. Pathway enrichment analysis was executed using the R package Piano v2.10.0 [30]. Pathways with p.adj(dist.dir.up) and p.adj(dist.dir.dn) < 0.05 were selected as significantly up-regulated and down-regulated pathways, respectively. KEGG pathway gene sets of categories including metabolism, environmental information processing, and organismal systems were used for the pathway enrichment analysis. Heatmaps were generated using the R package ComplexHeatmap v2.10.0 [31], and volcano plots were created using the R package ggplot2 v3.3.5. The Venn diagram was created using the online tool InteractiVenn [32], and the network visualization was created in Cytoscape v3.6.1.

### Gene expression analysis of SARS-CoV-2 host entry receptors and inflammatory mediators in astrocytes and pericytes

Astrocytes and pericytes were harvested at the designated time points (Fig. 1). RNA was isolated from the cells using QIAGEN RNeasy Plus Kits (cat #74034) as described previously [33], the messenger RNA (mRNA) was quantified using SimpliNano spectrophotometer (GE Healthcare Bio-Sciences Corp., Piscataway, NJ, USA) and stored in a − 80°C deep freezer for downstream analysis. 200 ng of RNA from each sample were converted into cDNA using SuperScript^™^ III First-Strand Synthesis SuperMix (catalog # 11752-050; ThermoFisher SCIENTIFIC) as per manufacturer’s instructions. The Quantitative RT-PCR assays were performed using TaqMan^®^ Gene Expression Assay (20 ) and TaqMan^™^ Universal PCR Master Mix (Catalog number: 4304437). The thermal cycling conditions were set to initial denaturation 95°C for 10 min, followed by 40 cycles of 95°C for 15 sec, 60°C for 1 min. The primer/probe set used in the study was as follows: GAPDH as endogenous control (Hs02758991_g1; VIC/MGB), ACE2 (Hs01085331_m1; FAM/MGB), TMPRSS2 (Hs00237175_m1; FAM/MGB), NRP1 (Hs00826128_m1; FAM/MGB), TRIM28 (Hs00232212_m1; FAM/MGB), IL-6 (Hs00174131_m1; FAM/MGB), TNF-α (Hs00174128_m1; FAM/MGB), IL-1β (Hs01555410_m1; FAM/MGB) and IL-18 (Hs01038788_m1; FAM/MGB). The fold change in gene expression values was calculated using the 2^−Δ(ΔCt)^ method; where ΔCt = Cttarget—Cthousekeeping and Δ(ΔCT) = ΔCt infected - ΔCt mock-infected.

## Results

### Abortive infection of SARS-CoV-2 in brain astrocytes and pericytes.

After 7 days post HIV infection, we measured the presence of HIV RNA in the culture supernatant of astrocytes and pericytes from all three donor cells in groups II and IV. We observed productive HIV replication in both astrocytes and pericytes, indicating active viral replication in both cell types (**Supplementary Fig. 1**). The cells from all four groups were infected with 1.0 MOI of SARS-CoV-2 (USA-WI1/2020) as described in Fig. 1.

**Figure 2:**
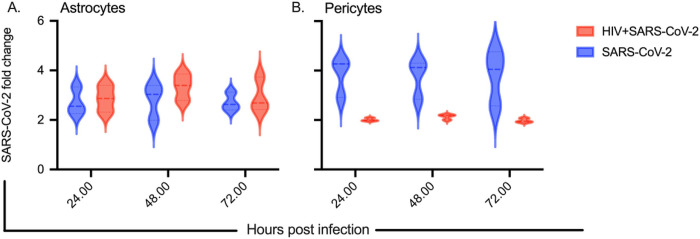
SARS-CoV-2 viral load in astrocytes and pericytes mono infected with SARS-CoV-2 or co-infected with HIV-1. The cells were infected with 1 MOI of SARS-CoV-2 and 0.1 MOI of HIV-1 in triplicate as described in Figure 1. Culture supernatant was collected after 1 h (considered as baseline), 24 h, 48 h and 72 h post SARS-CoV-2 infection. Fold change in SARS-CoV-2 viral load relative to baseline levels at 24, 48 and 72 h post infection in SARS-CoV-2 or HIV-1/SARS-CoV-2 co-infected astrocytes **(A)** and pericytes **(B).** Astrocytes and pericytes from three healthy donors were used in all experiments.

Using RT-qPCR, we measured the levels of SARS-CoV-2 RNA in the culture supernatant. The viral RNA copies at 1-hour post-infection were considered as baseline, and the fold change in RNA copy numbers at 24 hours, 48 hours, and 72 hours post-infection is depicted in Fig. 2A for astrocytes and in Fig. 2B for pericytes, respectively. In normal astrocytes infected with SARS-CoV-2, we observed a 2.7-fold increase at 24 hours, a 2.8-fold increase at 48 hours, and a 2.7-fold increase at 72 hours in mean viral genomic RNA copies from the three donors. In HIV-infected astrocytes, we detected a 2.8-fold increase at 24 hours, a 3.3-fold increase at 48 hours, and a 2.9-fold increase at 72 hours in mean viral genomic RNA copies from the three donors. In normal pericytes, we observed a 3.8-fold increase at 24 hours, a 3.7-fold increase at 48 hours, and a 3.7-fold increase at 72 hours in mean viral genomic RNA copies from the three donors. In HIV-infected pericytes, we measured a 2.0-fold increase at 24 hours, a 2.1-fold increase at 48 hours, and a 1.97-fold increase at 72 hours in mean viral genomic RNA copies from the three donors. Collectively, these findings indicate a restricted viral infection of brain astrocytes and pericytes in our experimental conditions.

### Proteomic analysis of primary brain astrocytes and pericytes

To investigate the potential link between SARS-CoV-2 infection and the development of neurocognitive disorders in HIV positive COVID-19 patients, we conducted proteomic analysis on astrocytes and pericytes infected with HIV, SARS-CoV-2, and HIV + SARS-CoV-2. Given the similar fold changes in viral replication at 24-, 48-, and 72-hours post SARS-CoV-2 infection, we focused on the 24-hour time point for the proteomic analysis. **Supplementary Figs. 2 and 3** depict volcano plots illustrating the differentially expressed proteins in mock vs. SARS-CoV-2, mock vs. HIV-1 + SARS-CoV-2, HIV-1 vs. HIV-1 + SARS-CoV-2, and SARS-CoV-2 vs. HIV-1 + SARS-CoV-2 infected astrocytes and pericytes, respectively.

**Figure 3:**
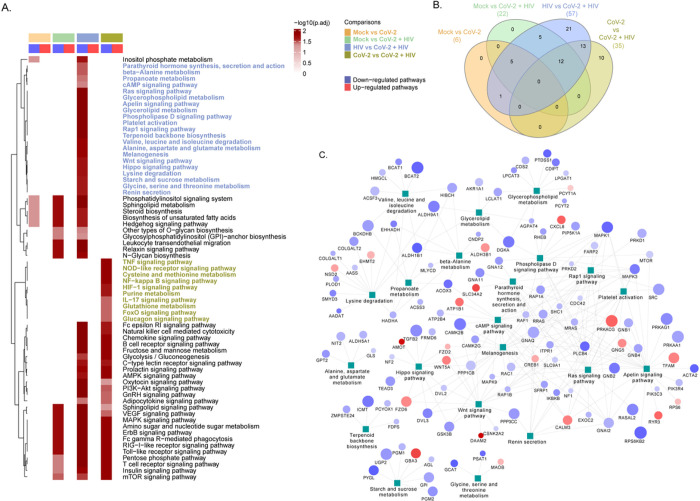
Comparative analysis of cellular pathways altered by differentially expressed proteins in SARS-CoV-2 and HIV-1/SARS-CoV-2 infected astrocytes. **(A)** Gene-set enrichment analysis results of the four pairwise comparisons namely, Mock vs SARS-CoV-2, Mock vs HIV-1/SARS-CoV-2, HIV-1 vs HIV-1/SARS-CoV-2, SARS-CoV-2 vs HIV-1/SARS-CoV-2. The heatmap visualizes negative log scaled adjusted p-values of all significantly up-regulated (p.adj<0.05) and down-regulated (p.adj<0.05) pathways. Column annotation denotes the comparisons and direction of regulation of pathways. **(B)** Venn diagram showing unique and common pathways significantly regulated (up/down) (p.adj<0.05) in each of the four pairwise comparisons shown in **Fig A. (C)** Network of significantly regulated (p.adj<0.05) proteins associated with uniquely regulated pathways in HIV-1 vs HIV-1+SARS-CoV-2. Protein-pathway associations are obtained from KEGG pathway gene-sets. Node size is relative to log scaled adjusted p-values of the proteins. Node color represents the direction of regulation of proteins, where downregulation of protein is denoted by blue color and up-regulation of protein is denoted by red color nodes. Astrocytes from three healthy donors were used in all experiments.

Due to restrictive SARS-CoV-2 infection in both cell types, we cannot detect any SARS-CoV-2 proteins in these cells. To gain insight into how the differentially regulated proteins may influence the host cellular function, we conducted gene-set enrichment analysis. The protein pathway associations were curated from KEGG pathway gene sets. Heat map of the significantly up-regulated and down-regulated pathways in astrocytes of the four pairwise comparisons is depicted in Fig. 3A. In astrocytes, the number of mutually inclusive and exclusive differentially regulated pathways observed in the pairwise comparison is shown in the Fig. 3B. In mock vs. SARS-CoV-2, there are 6; in mock vs. HIV-1 + SARS-CoV-2, there are 22; in HIV-1 vs. HIV-1 + SARS-CoV-2, there are 57; and in SARS-CoV-2 vs. HIV-1/SARS-CoV-2 there are 35 differentially regulated pathways detected. The network of significantly regulated (p.adj < 0.05) proteins associated with highly enriched pathways in HIV vs HIV + SARS-CoV-2 infected cells is shown in Fig. 3C. In this protein-protein interaction map, we observed several overlapping in the involvement of proteins between the significantly enriched pathways.

**Figure 4:**
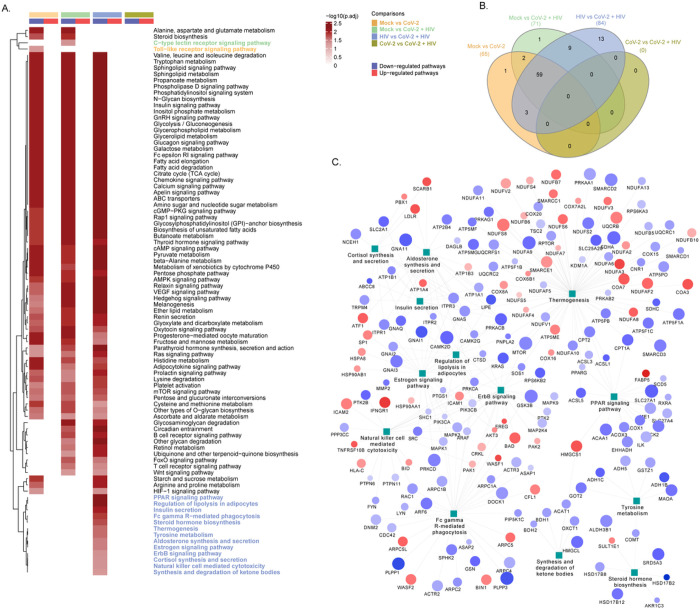
Comparative analysis of cellular pathways altered by differentially expressed proteins in SARS-CoV-2 and HIV-1/SARS-CoV-2 infected pericytes. **(A)** Gene-set enrichment analysis results of the four pairwise comparisons namely, Mock vs SARS-CoV-2, Mock vs HIV-1/SARS-CoV-2, HIV-1 vs HIV-1/SARS-CoV-2, SARS-CoV-2 vs HIV-1/SARS-CoV-2. The heatmap visualizes negative log scaled adjusted p-values of all significantly up-regulated (p.adj<0.05) and down-regulated (p.adj<0.05) pathways. Column annotation denotes the comparisons and direction of regulation of pathways. **(B)** Venn diagram showing unique and common pathways significantly regulated (up/down) (p.adj<0.05) in each of the four pairwise comparisons shown in **Fig A. (C)** Network of significantly regulated (p.adj<0.05) proteins associated with uniquely regulated pathways in HIV-1 vs HIV-1/SARS-CoV-2. Protein-pathway associations are obtained from KEGG pathway gene-sets. Node size is relative to log scaled adjusted p-values of the proteins. Node color represents direction of regulation of proteins, where downregulation of protein is denoted by blue color and up-regulation of protein is denoted by red color nodes. Pericytes from three healthy donors were used in all experiments.

Like astrocytes, the heat map of the significantly up-regulated and down-regulated pathways in pericytes of the four pairwise comparisons are depicted in Fig. 4A. The mutually inclusive and exclusive differentially regulated pathways observed in the pairwise comparison of the four groups is shown in the Fig. 4B. In mock vs. SARS-CoV-2, there are 65; in mock vs. HIV-1 + SARS-CoV-2 there are 71; in HIV-1 vs HIV-1 + SARS-CoV-2, there are 84; and in SARS-CoV-2 vs. HIV-1 + SARS-CoV-2 there are none differentially regulated pathways detected. The network of significantly regulated (p.adj < 0.05) proteins associated with uniquely regulated pathways in HIV vs HIV + SARS-CoV-2 is represented Fig. 4C. In this protein-protein interaction map, we observed several overlapping in the involvement of proteins between the significantly regulated pathways.

**Figure 5:**
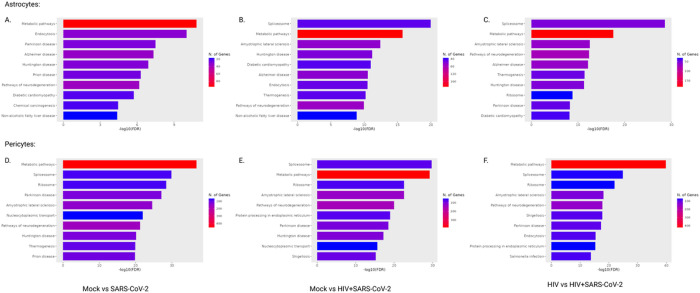
Gene ontology analysis of top ten differentially enriched pathways in astrocytes and pericytes. **(A)** Top ten enriched pathways in mock vs SARS-CoV-2 infected astrocytes. **(B)** Top ten enriched pathways in mock vs HIV+SARS-CoV-2 infected astrocytes. **(C)** Top ten enriched pathways in HIV vs HIV+SARS-CoV-2 infected astrocytes. **(D)** Top ten enriched pathways in mock vs SARS-CoV-2 infected pericytes. **(E)** Top ten enriched pathways in mock vs HIV+SARS-CoV-2 infected pericytes. **(F)** Top ten enriched pathways in HIV vs HIV+SARS-CoV-2 infected pericytes.

Finally, we performed a gene ontology enrichment analysis to find top ten enriched pathways using the differentially up or down regulated proteins with p.adj < 0.05, for all the pairwise comparations between four groups for astrocytes and pericytes using ShinyGO 0.77 [34]. In astrocytes, out of all pairwise comparisons, in mock vs. SARS-CoV-2, mock vs. HIV + SARS-CoV-2 and in HIV vs. HIV + SARS-CoV-2, we observed enrichment of neurological disorder pathways that includes Parkinson disease, Alzheimer disease, Huntington disease, and Amyotrophic lateral sclerosis as shown in Fig. 5A, B, and C. Similarly, in pericytes, out of all pairwise comparisons, in mock vs. SARS-CoV-2, mock vs. HIV + SARS-CoV-2 and in HIV vs. HIV + SARS-CoV-2, we observed enrichment of neurological disorder pathways that includes Parkinson disease, Huntington disease, and Amyotrophic lateral sclerosis as shown in Fig. 5D, E, and F respectively. Taken together this data indicates that SARS-CoV-2 alone or in presence of HIV imprint a proteomic signature in the astrocytes and pericytes linked to development of neurological disorders.

### Gene expression analysis of host cell SARS-CoV-2 entry receptors and inflammatory factors in astrocytes and pericytes

**Figure 6:**
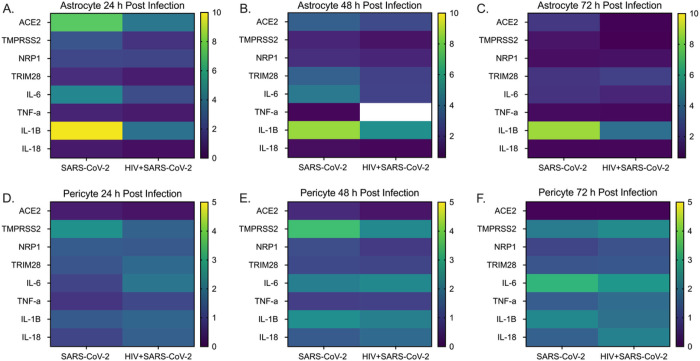
Differential gene expression analysis of SARS-CoV-2 host cell entry receptors and inflammatory factors in SARS-CoV-2 and HIV-1/SARS-CoV-2 infected astrocytes and pericytes compared to uninfected controls. SARS-CoV-2 and HIV-1/SARS-CoV-2 infected astrocytes 24 h **(A)**, 48 h **(B)** and 72 h **(C)** post SARS-CoV-2 infection. SARS-CoV-2 and HIV-1/SARS-CoV-2 infected pericytes 24 h **(D)**, 48 h **(E)** and 72 h **(F)** post SARS-CoV-2 infection. Astrocytes and pericytes from three healthy donors were used in all experiments.

Next, we examine the expression pattern of host cell receptors involved in SARS-CoV-2 entry and inflammatory factors in astrocytes and pericytes infected with SARS-CoV-2 and HIV + SARS-CoV-2. We assessed the changes in expression at 24, 48, and 72 h post infection compared to uninfected controls and represented the fold change in a heat map (Fig. 6: Astrocytes: 6A 24 h, 6B 48 h and 6C 72 h; Pericytes: 6D 24 h, 6E 48 h and 6F 72 h).

In SARS-CoV-2 infected astrocytes, the expression of ACE2 peaks at 24 h post-infection with a 7.4-fold increase. It then gradually decreased to 3.4-fold and 2.1-fold change at 48 h and 72 h, respectively. In HIV + SARS-CoV-2 infected astrocytes, ACE-2 expression showed a 4.0-fold increase at 24 h, followed by a decrease to 2.7-fold and 0.6-fold change at 48 h and 72 h, respectively. The expression of TMPRSS2, NRP1, and TRIM28 the other host cell entry factors, also showed modest increases in both groups at all time points. Regarding inflammatory mediators, IL-6 expression showed a 4.6-fold, 4.4-fold, and 2.0-fold increase in SARS-CoV-2 infected astrocytes, and a 2.3-fold, 2.4-fold, and 1.6-fold increase in HIV + SARS-CoV-2 infected astrocytes at the respective time points. Similarly, IL-1β expression increased by 9.8-fold, 8.6-fold, and 8.6-fold in SARS-CoV-2 infected astrocytes, and by 3.7-fold, 5.3-fold, and 3.9-fold in HIV + SARS-CoV-2 infected astrocytes at the respective time points.

In pericytes, we observed a decrease in ACE2 expression in both SARS-CoV-2 and HIV + SARS-CoV-2 infected cells at all time points. We observed a modest increase in the expression of TMPRSS2, NRP1, TRIM28, IL-6, TNF-α, IL-1β and IL-18 in both the groups at all the time points.

In summary, our analysis revealed a modest increase in the expression of SARS-CoV-2 host cell entry receptors and pro-inflammatory mediators in astrocytes, particularly at 24 hours post-infection. Notably, although the expression of the tested genes was generally higher in the SARS-CoV-2 group compared to the HIV + SARS-CoV-2 group, the differences were not statistically significant. The changes in pericytes were not as pronounced as those observed in astrocytes for both groups of infected cells.

## Discussion

Neurocognitive impairments associated with COVID-19 are increasing, both during acute infection and in post-acute sequelae of COVID-19 (PACS). The impact of SARS-CoV-2 infection in the central nervous system (CNS) of people living with HIV (PWH), who already have compromised blood-brain barrier (BBB) and persistent brain inflammation, is not well studied. To address this, we investigated the consequences of HIV/SARS-CoV-2 coinfection in primary human astrocytes and pericytes, which are major targets of SARS-CoV-2. We found that SARS-CoV-2 replication was limited in healthy and HIV-infected astrocytes and pericytes. SARS-CoV-2 Mono and co-infection with HIV resulted in a modest increase in the expression of viral host cell entry factors and pro-inflammatory mediators like IL-6 and IL-1β in astrocytes. Using quantitative proteomic analysis, we identified a distinct protein expression signature in co-infected astrocytes/pericytes compared to healthy, SARS-CoV-2, or HIV-infected cells. The regulated pathways in co-infected cells are associated with the development of neurological disorders such as PD, AD, HD, and ALS. Several symptoms of these diseases are frequently observed in neuro-PASC patients [35]. In addition, these findings have critical implications for PWH, as they frequently experience symptoms resembling these neurological disorders [36].

Viral infections manipulate host cell machinery for their survival and immune evasion [37]. The expression of host cell entry factors plays an essential role in establishing and persistence of viral infection. We examined the expression of SARS-CoV-2 entry receptors in astrocytes and pericytes infected with the virus alone or in combination with HIV. Our findings align with previous studies including ours, showing low to moderate expression of ACE2 and NRP1 in healthy and SARS-CoV-2 infected brain cells [38–41]. Research using brain tissue from COVID-19 patients suggests that ACE2-expressing pericytes may facilitate SARS-CoV-2 entry into the brain [18]. Similarly, in cortical assembled models, pericyte-like cells expressing ACE2 serve as sites for viral replication [42]. Furthermore, studies on human monocyte derived macrophages have shown that restricted SARS-CoV-2 infection triggers dysregulated immune responses and overexpression of pro-inflammatory cytokines [43, 44]. Taken together, these findings support our observations that restrictive SARS-CoV-2 infection in healthy and HIV-infected astrocytes and pericytes leads to a modest increase in inflammatory mediator expression, potentially disrupting normal brain function.

Many people after recovery forms of acute COVID-19 are suffering from PASC with cognitive impairments, and atrophy in the orbitofrontal and the para-hippocampal regions [45, 46]. These symptoms are similar to HAND observed in PWH [47]. Therefore, it is important to understand the long- term impact of COVID-19 in PWH. There is an ongoing argument on the mechanism of COVID-19-associated neuropathogenesis (i) Is it the result of direct invasion of SARS-CoV-2 in the brain or (ii) the consequence of systemic changes due to infection? Current data demonstrates a heterogeneous distribution of SARS-CoV-2 genomic RNA and viral proteins in brain even after recovery from active disease, and the underlying molecular mechanism remains unknown [42, 48–51]. Our findings indicate that even without establishing a productive infection, SARS-CoV-2 can induce a distinct proteomic signature in both healthy and HIV-infected astrocytes and pericytes.

In a Healthy brain, astrocytes perform various functions such as producing and releasing growth factors like NGF, BDNF, FGF-2, PDGF, GDNF, and TGFβ [52], maintaining BBB integrity, regulating cerebral blood flow, and water homeostasis, serving as an energy source for neurons, and maintaining the balance of glutamate-glutamine levels [53, 54]. However, during a pathogenic insult, in astrocytes undergo morphological, transcriptional, and functional changes, contributing to the development of neurodegenerative disorders [55]. Additionally, studies have shown that astrocytes can act as reservoirs for HIV reservoirs with restrictive replication and efficient cell-to-cell transmission capability [56, 57]. HIV infection induces activation of astrocytes, leading to the release of pro-inflammatory cytokine/chemokines and glutamate, causing excitotoxicity and neuronal death [58].

In our study, we observed differential modulation of energy metabolism pathways, such as Glycolysis/Gluconeogenesis and the Pentose phosphate pathway (PPP), in HIV/SARS-CoV-2 co-infected astrocytes. Dysregulation PPP has been linked to the development of neurodegenerative disorders line PD, AD, and ALS [59]. We also found enrichment of pathways related to propanoate metabolism, which is interconnected with beta-Alanine metabolism, and has been associated with AD development [60]. Additionally, we detect alteration in glycerophospholipid metabolism, that can impact anxiety-like behaviors, locomotor activity [61], and α-Synuclein mediated PD pathogenesis [62]. Astrocytes convert glutamate to glutamine, which helps maintain a glutamate/GABA balance in synaptic clefts and maintains the equilibrium in glutamatergic/GABAergic neurotransmission in CNS which is detrimental for its function [63]. HIV or its proteins like TAT/gp120 disrupt the Glu/GABA/Gln balance resulting in several neurodegenerative disorders [64]. Therefore, dysregulation in alanine-aspartate-glutamate metabolism in astrocytes of COVID-19 patients living with HIV may potentiate the development of neurocognitive disorders. Again, AD is associated with the dysregulation of several amino acid metabolisms [65] and we also detected enrichment of several amino acid metabolisms and degradation pathways in HIV/SARS-CoV-2 co-infected astrocytes. These findings suggest that PWH should be closely monitored for the development of AD-associated symptoms even after recovering from acute COVID-19 infection.

Pericytes are multifunctional mural cells surrounded by the basement membrane endothelial cells of the blood vessels throughout the body [66]. In the brain, pericytes remain embedded between brain microvascular endothelial cells, astrocytes, and neurons. They control BBB permeability, regulate angiogenesis and transmigration of systemic leukocytes in CNS, clear tissue debris, and Alzheimer’s amyloid-β (Aβ) toxin from CNS [67]. Disruption of signaling pathways in pericytes can contribute CNS disorders like AD, and ALS [68–71]. Recent research suggests that pericytes also support productive HIV replication [72, 73]. Productive infection or exposure to HIV proteins results in the loss of pericytes from the BBB tight junction, which increases its permeability and facilitates the transmigration of systemic leukocytes in the brain parenchyma. This also leads to overexpression of pro-inflammatory mediators like IL-6, TNF-α, and IL-1β in pericytes, that triggers neuroinflammation [74, 75].

We identified specific cellular pathways that were differentially regulated in pericytes co-infected with HIV and SARS-CoV-2. These pathways include steroid hormone biosynthesis, thermogenesis, estrogen signaling, and ErbB signaling, which are all important for normal brain function [76]. Dysregulation in these pathways can have negative effects on neurogenesis, memory function, energy balance, and mental health [77]. Again, alteration in ErbB signaling in the brain, as observed in co-infected pericytes, is related to the development of depression [78]. This finding is also supported by the fact that many COVID-19 patients living with HIV develop depression after recovery from acute infection [79]. Mallat et al. describe the spike protein of SARS-CoV-2 damaging the vascular and immune regulatory functions of pericytes, resulting in vascular-mediated brain damage [21]. We also observed several immune-related pathways uniquely regulated in HIV/SARS-CoV-2 co-infected pericytes like Fc gamma receptor-mediated phagocytosis pathway, NK-cell mediated cytotoxicity pathways.

The limitation of the study includes the use of primary human astrocytes and pericytes. The human brain is a complex organ built with numerous cell types, which are interlinked with each other to form a sophisticated network that works in unison. The neurological consequence of HIV/SARS-CoV-2 may be a result of direct viral invasion to the CNS and neuronal injury, or indirect impact of systemic infection, involvement of a neuro-immune response, inflammation and immune activation, post-infection autoimmunity, and neurovascular injury, which is challenging to study using individual cell types [47, 80]. But no small animal model is available to study the impact of HIV/SARS-CoV-2 in the CNS, and the cerebral organoids used to study the SARS-CoV-2 associated neurological consequences are not susceptible to HIV infection. Other factors may impact the neurological outcomes, like whether the patients are on combined antiretroviral therapy or have complete plasma viral suppression, etc., which warrants the development of biologically relevant animal model to study in detail.

## Conclusions

To the best of our knowledge this is the first study reporting the impact of HIV/SARS-CoV-2 co-infection in two different CNS primary cell types. Our findings suggest that astrocytes and pericytes only support abortive SARS-CoV-2 replication in healthy or HIV-infected cells. The co-infection in the astrocytes and pericytes impart a unique proteomic signature corresponding to several cellular signaling pathways linked to energy metabolism, amino acid metabolism, and degradation. Several neurodegenerative diseases are associated with these differentially modulated pathways that, include but are not limited to PD, AD, Huntington Disease and ALS. Therefore, our study suggests the importance of long-term monitoring HIV/SARS-CoV-2 co-infected patients for possible development of neurodegenerative disorders.

## Data Availability

The mass spectrometry proteomics data have been deposited to the ProteomeXchange Consortium via the PRIDE partner repositor. Rest of all data generated in this study are included in this manuscript.

## Abbreviations

ACE2: Angiotensin converting enzyme 2
AD: Alzheimer’s Disease
ALS: Amyotrophic lateral sclerosis
Aβ: amyloid-β
BBB: Blood Brain Barrier
CNS: Central nervous system
COVID-19: Coronavirus disease of 2019
EMEM: Eagle’s Minimum Essential Medium
HAND: HIV-Associated Neurocognitive Disorder
HD: Huntington Disease
HIV: Human immunodeficiency virus
IL-1b: Interleukin 1 beta
IL-18: Interleukin 18
IL-6: Interleukin 6
MDM: Monocyte-derived macrophages
MERS-CoV: Middle East respiratory syndrome coronavirus
MOI: Multiplicity of infection
NRP1: Neuropilin 1
PASC: Post-acute sequelae of COVID-19
PD: Parkinson’s Disease
PNS: Peripheral nervous system
PWH: People living with HIV
SARS-CoV: Severe acute respiratory syndrome coronavirus
SARS-CoV-2: Severe acute respiratory syndrome coronavirus 2
snRNA-seq: Single-nucleus RNA sequencing
TMPRSS2: Transmembrane serine protease 2
TNF-a: tumor necrosis factor alpha
TRIM28: Tripartite motif containing 28
